# Impact of *APOL1* Genetic Variants on HIV-1 Infection and Disease Progression

**DOI:** 10.3389/fimmu.2019.00053

**Published:** 2019-01-24

**Authors:** Ping An, Gregory D. Kirk, Sophie Limou, Elizabeth Binns-Roemer, Jeffrey B. Kopp, Cheryl A. Winkler

**Affiliations:** ^1^Molecular Genetic Epidemiology Section, Basic Science Program, Basic Research Laboratory, Frederick National Laboratory for Cancer Research, National Cancer Institute, Frederick, MD, United States; ^2^Departments of Epidemiology and Medicine, Johns Hopkins University, Baltimore, MD, United States; ^3^CRTI UMR1064, Inserm, Université de Nantes & ITUN, CHU Nantes, Nantes, France; ^4^Ecole Centrale de Nantes, Nantes, France; ^5^Kidney Disease Section, National Institute of Diabetes and Digestive and Kidney Diseases, NIH, Bethesda, MD, United States

**Keywords:** HIV-1, AIDS, APOL1, host susceptibility, genetic epidemiology

## Abstract

Apolipoprotein L1 (APOL1) has broad innate immune functions and has been shown to restrict HIV replication *in vitro* by multiple mechanisms. Coding variants in *APOL1* are strongly associated with HIV-associated nephropathy (HIVAN) in persons with untreated HIV infection; however, the mechanism by which *APOL1* variant protein potentiates renal injury in the presence of high viral load is not resolved. Little is known about the association of *APOL1* genotypes with HIV viral load, HIV acquisition, or progression to AIDS. We assessed the role of *APOL1* coding variants on HIV-1 acquisition using the conditional logistic regression test, on viral load using the *t-*test or ANOVA, and on progression to AIDS using Cox proportional hazards models among African Americans enrolled in the ALIVE HIV natural history cohort (*n* = 775). *APOL1* variants were not associated with susceptibility to HIV-1 acquisition by comparing genotype frequencies between HIV-1 positive and exposed or at-risk HIV-1 uninfected groups (recessive model, 12.8 vs. 12.5%, respectively; OR 1.02, 95% CI 0.62–1.70). Similar null results were observed for dominant and additive models. *APOL1* variants were not associated with HIV-1 viral load or with risk of progression to AIDS [Relative hazards (RH) 1.33, 95% CI 0.30–5.89 and 0.96, 95% CI 0.49–1.88, for recessive and additive models, respectively]. In summary, we found no evidence that *APOL1* variants are associated with host susceptibility to HIV-1 acquisition, set-point HIV-1 viral load or time to incident AIDS. These results suggest that APOL1 variants are unlikely to influence HIV infection or progression among individuals of African ancestry.

## Introduction

Apolipoprotein L1 (APOL1) is a human innate immune factor against African trypanosomes responsible for human African trypanosomiasis (or sleeping sickness) (1). Two common *APOL1* variants, G1 (rs73885319, p.S342G) and G2 (a 6-bp in-frame deletion removing two amino acids, rs71785313, p.N388_Y389del), extend APOL1 restriction to *T.b.rhodesiense*, the cause of acute human African trypanosomiasis. These variants are found only in individuals with recent African ancestry. The 12–14% of African Americans carrying two *APOL1* renal risk alleles in the compound heterozygous or homozygous state (referred to as *APOL1* high risk [HR] genotypes) have a 3-, 7-, and 17-fold increased risk for developing hypertension-attributed nephropathy, non-diabetic end-stage kidney disease, and focal segmental glomerulosclerosis, respectively, (2–4). *APOL1* is most strongly associated with HIV-associated nephropathy (HIVAN), with odds ratio (OR) 29 in African Americans and OR 89 in South Africans (3, 5), suggesting a strong interaction between APOL1 and the HIV-1 virus. The lifetime risk of HIVAN, a form of collapsing focal segmental glomerulosclerosis associated with rapid progression to end-stage renal disease, is ~10% in African Americans with untreated HIV infection (6, 7). The pathogenesis of HIVAN is likely due to direct HIV infection of kidney epithelial cells, which leads to podocyte proliferation and APOL1-mediated podocyte injury and loss (8–11). *APOL1* transcription is up-regulated by interferons and other pro-inflammatory cytokines (12).

Recently, Taylor et al. reported that APOL1 restricts HIV-1 replication in macrophages and differentiated monocytes (12). APOL1 was shown to target HIV-1 Gag for degradation by the endolysosomal pathway and to deplete HIV-1 Vif, which counteracts the APOBEC3G host restriction factor in lysosomes (12). However, it remains unknown if variant APOL1 affects HIV acquisition, viral replication, or HIV disease progression.

*APOL1* renal risk variants are most common in West Africa, where the prevalence of *APOL1* HR genotypes approaches 25% but are also found throughout sub-Saharan Africa (4, 13) where HIV-1 infection is notably prevalent. Although *APOL1* renal risk variants are a risk factor for kidney disease in HIV-1 infected persons, it is unknown if *APOL1* renal risk variants are associated with other HIV-1 phenotypes. In the present study, we evaluate the genetic associations between *APOL1* variants and HIV-1 acquisition, set-point viral load, and rate of progression to AIDS among African Americans enrolled in the ALIVE HIV-1 cohort.

## Materials and Methods

### Ethics Statement

Ethical approval for the study was obtained from the National Institute of Health Office of Human Subjects Research Protections (OHSRP #3314). Review Boards of participating institutions approved the study protocols, and written informed consent was obtained from all study participants.

### Study Participants

Since *APOL1* G1-G2 alleles are found only on African-origin chromosomes, we studied only African American participants enrolled in the ALIVE (AIDS Link to the Intravenous Experience) cohort.

### The ALIVE Cohort

The epidemiological and clinical characteristics of the ALIVE cohort have been previously described (14). ALIVE is a prospective longitudinal natural cohort originally designed to characterize the incidence and natural history of HIV infection among injection drug users (IDUs) in Baltimore, MD, initiated in 1988 (14). At study entry, 88% of participants were African Americans. The participants were followed up semi-annually with blood draws for viral load and CD4+ T cell measurements and physical exam at each visit.

The study group comprises 227 African American incident HIV-1 seroconverters, 213 HIV-1 seroprevalent participants, and 335 uninfected, IDU participants. Seroconversion date was estimated as the midpoint between the last seronegative and the first seropositive HIV-1 antibody test date (mean interval 0.66 years, range 0.11–3.4 years) (15).

### Genotyping of APOL1 G1-G2 Risk Variants

*APOL1* renal risk variants G1 (rs73885319, p.S342G) and G2 (rs71785313, p.N388_Y389del) were genotyped by TaqMan genotyping assays (Applied Biosystems, Foster City, CA). The TaqMan allele discrimination assays were carried out on an ABI 7900HT sequencer detector system (Applied Biosystems, Foster City, CA, USA), according to the manufacturer's protocol as previously described (3). For quality control, water controls were included on each plate and 10% of samples were duplicated. No water contamination or genotype mismatches between duplicates was observed. The genotype results were also further validated by the Sanger sequencing, following the protocol previously described (16).

### Defining APOL1 Risk Haplotypes

The G1 risk allele is defined by the presence of the G allele at rs73885319 (342G) and the G2 (6-del) risk allele by the 6 base pair deletion at rs71785313 (-/TTATAA), which leads to the loss of two amino acids (388–389NYK>K) (2, 3). The G1 and G2 risk alleles are in absolute negative disequilibrium and never occur together on the same chromosome (17)). *APOL1* follows a recessive inheritance model for HIVAN and other kidney diseases: *APOL1* HR status for kidney disease is defined by carriage of 2 risk alleles (G1/G1, G1/G2, and G2/G2) and low-risk (LR) status is defined by carriage of 1 or 0 renal risk alleles (18).

### Statistical Analysis

We assessed the potential effects of *APOL1* risk genotypes using additive (2 vs. 1 vs. 0 risk alleles), dominant (2 or 1 vs. 0), and recessive (2 vs. 1 or 0 risk alleles) models. All analyses were performed using SAS version 9.12 (SAS Institute, Cary, NC).

### Analysis of Risk to HIV-1 Acquisition

We assessed the impact of *APOL1* G1-G2 variants on HIV-1 infection susceptibility by comparing frequencies between the HIV-1 infected group comprising HIV-1 seroincident and seroprevalent subjects and the HIV-1 at-risk, uninfected group. Odds ratios (OR) and *P*-values were obtained by using a conditional logistic regression test. Statistical power was calculated with GAS-power-calculator available at http://csg.sph.umich.edu/abecasis/gas_power_calculator/.

### Analysis of Viral Load

For the seroincident participants, HIV-1 viral load set-point was defined as the mean log_10_-transformed HIV-1 RNA plasma copies measured between 6 and 33 months after seroconversion (2–5 measurements). Viral load measurements exceeding 3-fold (0.5 log_10_) from the average of all remaining points were excluded, as previously suggested (19). We ran *t*-tests to estimate the difference of viral load means between *APOL1* HR and LR subgroups. We used the one-way analysis of variance (ANOVA) to determine whether there were any statistically significant differences among the means for carriage of 2, 1, or 0 *APOL1* risk alleles.

### Analysis of Disease Progression to AIDS

In the ALIVE cohort, we tested the association of *APOL1* risk alleles on disease progression to AIDS using Cox proportional hazards model (Cox model) and Kaplan-Meier survival curve analyses for incident HIV seroconverters. The disease progression endpoints were: CD4 T-cell <200 cells/mm^3^, or clinical AIDS diagnosis (20). The median time from seroconversion to AIDS was 7 years. To avoid the confounding effect of anti-retroviral therapy (ART) on disease progression, we censored the data after July 31, 1997 as few ALIVE participants received ART prior to this date (21). We included known genetic factors modifying AIDS progression as covariates in the adjusted Cox model analysis: *HLA*-B^*^57 and *HLA* Class I homozygosity (22). The analyses were stratified by sex and by age at seroconversion: 0–20, 20–40, and >40 years. Two-tailed *P*-values were computed using Wald tests.

## Results

### Association of *APOL1* Risk Alleles on the Risk of HIV-1 Acquisition

To determine whether *APOL1* G1 or G2 variants affect host susceptibility to HIV-1 acquisition, we compared the distribution of G1 and G2 variants in HIV-1 seroincident subjects (*n* = 227) with at risk, seronegative individuals (*n* = 335) (Table 1). No associations with HIV-1 acquisition were observed for the additive (*P* = 0.61), dominant (*P* = 0.56) or recessive genetic models (*P* = 0.87) (Table 1). To increase power, we combined seroconverters and seroprevalents) but results remained non-significant (Table 1). Adjusting for sex and age did not affect the results (Table 1). These results suggest that *APOL1* risk variants have no impact on host susceptibility to HIV-1 acquisition.

**Table 1:** Association of APOL1 G1-G2 variants with HIV-1 acquisition.

**HIV-1 status**	***n***	**Age (years)$**	**Female& (%)**	**No. G1-G2 risk alleles**			**Comparison**	Model^*^	OR (95% CI)^*^	P^*^
				**0**	**1**	**2**			
Seroincident (SI)	227	39.9 ± 6.3	25.1	88 (38.8%)	110 (48.5%)	29 (12.8%)	SI vs. SN	Add	1.07 (0.84–1.38)	0.57
								Add_adj_	1.07 (0.83–1.37)	0.61
								Dom	1.13 (0.80–1.60)	0.47
								Dom_adj_	1.10 (0.78–1.57)	0.56
								Rec	1.02 (0.62–1.70)	0.98
								Rec_adj_	1.04 (0.63–1.74)	0.87
Seroincident (SI) + seroprevalent (SP)	441	40.5 ± 6.2	25.0	177 (41.8%)	217 (49.2%)	47 (10.7%)	SI+SP vs. SN	Add	0.99 (0.80–1.23)	0.96
								Add_adj_	1.00 (0.81–1.24)	0.99
								Dom	1.07 (0.80–1.43)	0.64
								Dom_adj_	1.07 (0.80–1.43)	0.64
								Rec	0.83 (0.54–1.30)	0.42
								Rec_adj_	0.84 (0.54–1.31)	0.45
Seronegative (SN)	335	41.5 ± 7.4	29.3	140 (41.8%)	153 (45.7%)	42 (12.5%)	Reference		-	-

*There were no associations between APOL1 kidney risk allele status and HIV-1-infection status. $P > 0.05 for SN vs. SI + SP; P < 0.05 for SN vs. SI; & P > 0.05 for SN vs. SI+SP or SN vs. SI*.

* *Logistic regression for additive (Add, 2 vs. 1 vs. 0), dominant (Dom, 2 or 1 vs. 0), and recessive (Rec, 2 vs. 1 or 0) genetic models, adjusted (adj) for age (years) and stratified on sex*.

### Association of *APOL1* Risk Alleles on HIV-1 Viral Load

Among HIV-1 seroincident participants, set-point HIV-1 viral load levels were found to be similar for carriers of 2, 1, or 0 *APOL1* risk alleles (*P* = 0.79, ANOVA). In the recessive model comparing viral load between carriers of HR genotypes (VL = 4.23 ± 0.64, *N* = 27) and those with LR APOL1 genotypes (VL = 4.20 ± 0.73, *N* = 177), we also observed no differences in viral load (*P* = 0.48, Table 2).

**Table 2:** Association between *APOL1* G1-G2 variant alleles and HIV-1 viral load.

**No. *APOL1* G1-G2 risk alleles**	***n***	**Viral load (SD)**	**Comparison**	P*
0 or 1	177	4.20± 0.73	ref	1
0	82	4.26 ± 0.68	ref	1
1	95	4.19 ± 0.77		0.52
2	27	4.23 ± 0.65		0.67
			Additive (2 vs. 1. vs. 0)	0.79
			Dominant (2 or 1 vs. 0)	0.50
			Recessive (2 vs. 1 or 0)	0.86

There were no associations between number of APOL1 kidney risk alleles and HIV-1 viral load, presented as log base 10, copies/ml. *From t-tests or ANOVA (additive); SD, Standard Deviation.

### Association of *APOL1* Risk Alleles on HIV-1 Disease Progression

**Figure 1:**
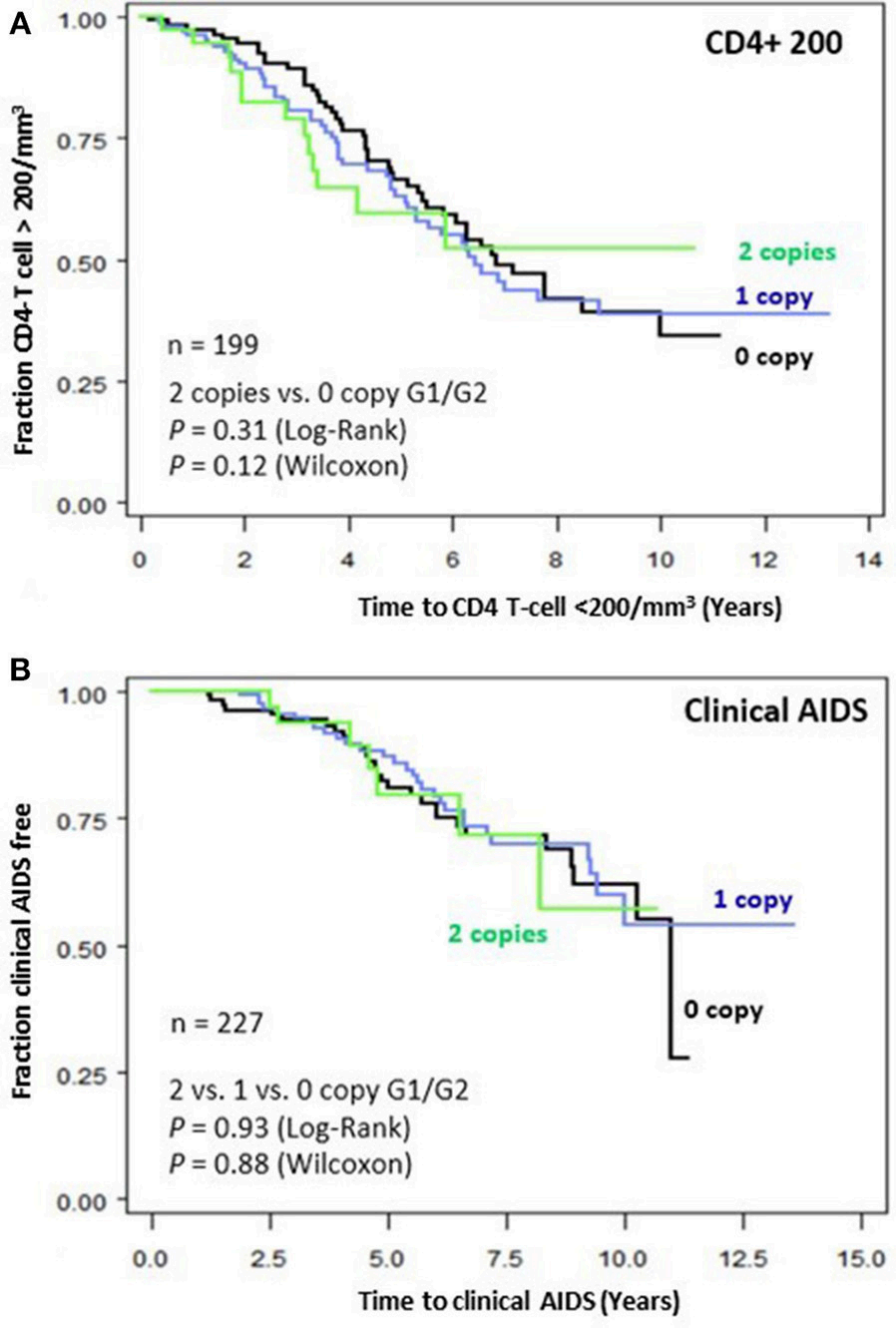
Genetic effects of APOL1 G1-G2 variants on progression of HIV disease. Kaplan-Meier survival curves for carriage of 0, 1, and 2 *APOL1* risk allele for progression to **(A)** CD4+ T-cell <200/mm^3^ and **(B)** clinical AIDS. RH and adjusted *P*-values were estimated from Cox proportional hazards models. *P*-values for survival curves were obtained from a log-rank test.

To assess the impact of *APOL1* HR on disease progression in untreated individuals from date of seroconversion to CD4 <200 cell/mm^3^ and to incident AIDS, we performed time-to-event analysis for 227 African American seroincident participants. *APOL1* genotypes were not associated with the rate of progression to CD4 <200 cells/mm^3^ (Figure 1A) or AIDS in Kaplan-Meier survival analyses (Figure 1B, additive model, *P* > 0.12 for log-rank or Wilcoxon tests). In the crude (data not shown) and adjusted Cox models, *APOL1* HR genotype (recessive model), *APOL1* risk allele number (additive) or risk allele carriage (dominant model) were not associated with the rate of progression to AIDS (*P* > 0.70, Table 3).

**Table 3:** Association between *APOL1* G1-G2 variant alleles and incident clinical AIDS.

**No. *APOL1* G1-G2 risk alleles**	***n***	**RH**	**95% CI**	***P***
0 (reference)	88	1	–	1
1 (1 vs. 0)	110	1.11	0.52–2.34	0.79
2 (2 vs. 0)	29	0.87	0.16–4.74	0.87
Additive (2 vs. 1. vs. 0)		1.09	0.59–2.02	0.79
Dominant (2 or 1 vs. 0)		1.07	0.51–2.24	0.85
Recessive (2 vs. 1 or 0)		1.24	0.28–5.40	0.78

There were no associations between APOL1 kidney risk alleles and incident AIDS. Cox model results, adjusted for age, sex, HLA-B^*^57, and HLA Class I homozygosity.

## Discussion

In this genetic epidemiological study of an HIV-1 at-risk and natural progression cohort, we observed no evidence of association between *APOL1* renal risk alleles and HIV-1 acquisition, HIV-1 viral load, and rate of progression to CD4 <200, AIDS, or the composite outcome. Our results indicate that *APOL1* renal risk variants, which are highly prevalent among African Americans and sub-Saharan Africans, do not significantly contribute to the HIV-1 epidemic by increasing viral burden or potentiating HIV-1 transmission.

A recent *in vitro* study reported that APOL1 protein can inhibit HIV-1 infection of macrophage and monocytes by multiple mechanisms, including inhibition of transcription and degradation of HIV-1 Gag and Vif proteins (12). If APOL1 protein effectively inhibits HIV-1 *in vitro, APOL1* coding variants might confer differential impact on HIV replication or disease progression by enhancing or attenuating the anti-HIV properties of APOL1 protein. However, our genetic association study revealed no *in vivo* evidence of association of *APOL1* renal risk alleles with HIV-1 infection acquisition or disease progression. Our findings are supported by the observation that *APOL1* gene expression is undetectable in CD4+ T cells, the primary target of HIV infection even with IFN-γ stimulation (12). In contrast, *APOL1* gene expression is highly inducible by IFN-γ stimulation in monocytes and macrophages, which were used in the *in vitro* experiments testing for APOL1 restriction of HIV replication (12). CD4^+^ T lymphocytes are the principal target of HIV, while infected macrophages play a supportive role in viral pathogenesis involving HIV cell-to-cell spread, and certain tissue infections including lungs, gut and brain (23, 24). The *in vivo* role of APOL1 in HIV-1 pathogenesis thus warrants further investigation. An implication of this study is that development of HIVAN and eGFR decline among those with *APOL1* HR status (18, 25, 26), is likely due to local podocyte injury in a setting of high viral load in patients with untreated HIV infection. A recent study demonstrated that variant APOL1 protein increases accumulation of HIV-1 in podocytes, inducing inflammatory responses via IL-1β priming (11).

This study has both strengths and limitations. A strength is that the ALIVE cohort is one of few well-characterized HIV natural history cohorts enrolling African Americans prior to the ART era, and the large number of treatment-naïve seroconverters makes it a choice cohort for unbiased exploration of HIV-related outcomes. The relatively modest sample size is balanced by the combined high frequency of these variants in the African American population. We had 80% power to detect a potential association of *APOL1* G1-G2 with HIV-1 infection, with an OR 1.35 for additive model and 1.93 for recessive model. We were unable to control for mortality due to *APOL1*-associated ESKD or to HIVAN since biopsy data were unavailable; however, only 1 death was observed among 29 APOL1 HR individuals prior to censoring on July 31, 1997, suggesting that our null results are not due to frailty bias resulting from excess HIVAN or ESKD-related deaths in the HR group.

In summary, this population genetic study found no evidence that *APOL1* renal risk variants contribute to the risk of HIV-1 acquisition or progression of HIV-1 disease progression to AIDS. *APOL1* variants are unlikely to contribute to the prevalence of HIV infection in subSaharan Africa or among African Americans.

## Author Contributions

PA and CW conceived the study, designed the analyses, and wrote the manuscript. PA performed the analyses. EB-R performed genotyping. GK provided clinical data and DNA samples. GK, SL, and JK contributed to data interpretation and manuscript revisions. All authors reviewed the manuscript.

### Conflict of Interest Statement

The authors declare that the research was conducted in the absence of any commercial or financial relationships that could be construed as a potential conflict of interest.

## Abbreviations

HIV-1: HIV type 1
OR: Odds ratio
RH: Relative Hazards
SNP: Single Nucleotide Polymorphism.
